# PLAC1 is essential for FGF7/FGFRIIIb-induced Akt-mediated cancer cell proliferation

**DOI:** 10.18632/oncotarget.27582

**Published:** 2020-05-19

**Authors:** Diana Barea Roldán, Matthias Grimmler, Christoph Hartmann, Stefanie Hubich-Rau, Tim Beißert, Claudia Paret, Giuseppe Cagna, Christoph Rohde, Stefan Wöll, Michael Koslowski, Özlem Türeci, Ugur Sahin

**Affiliations:** ^1^TRON–Translational Oncology at the University Medical Center of the Johannes Gutenberg University Mainz, Mainz, Germany; ^2^Formerly of TRON–Translational Oncology at the University Medical Center of the Johannes Gutenberg University Mainz, Mainz, Germany; ^3^Formerly of Ganymed Pharmaceuticals AG, Mainz, Germany; ^4^Biopharmaceutical New Technologies (BioNTech) Corporation, Mainz, Germany; ^5^Formerly of University Medical Center of the Johannes Gutenberg University Mainz, Mainz, Germany; ^6^University Medical Center of the Johannes Gutenberg University Mainz, Mainz, Germany; ^7^Ci3 Cluster for Individualized Immune Intervention, Mainz, Germany; ^8^Current address: DiaSys Diagnostic Systems GmbH, Holzheim, Germany; ^9^Current address: Merck KGaA, Darmstadt, Germany; ^10^Current address: Lonza Pharma & Biotech, Cologne, Germany; ^11^Current address: Merck KGaA, Darmstadt, Germany; ^12^Current address: GammaDelta Therapeutics, London, United Kingdom; ^*^These authors contributed equally to this work

**Keywords:** PLAC1, tumorigenesis, extracellular matrix, fibroblast growth factor, AKT

## Abstract

PLAC1 (placenta enriched 1) is a mammalian trophoblast-specific protein. Aberrant expression of PLAC1 is observed in various human cancers, where it is involved in the motility, migration, and invasion of tumor cells, which are associated with the phosphoinositide 3-kinase (PI3K)/AKT pathway. We previously demonstrated that AKT activation mediates the downstream effects of PLAC1; however, the molecular mechanisms of PLAC1-induced AKT-mediated tumor-related processes are unclear. We studied human choriocarcinoma and breast cancer cell lines to explore the localization and receptor-ligand interactions, as well as the downstream effects of PLAC1. We show secretion and adherence of PLAC1 to the extracellular matrix, where it forms a trimeric complex with fibroblast growth factor 7 (FGF7) and its receptor, FGF receptor 2 IIIb (FGFR2IIIb). We further show that PLAC1 signaling via FGFR2IIIb activates AKT phosphorylation in cancer cell lines. As the FGF pathway is of major interest in anticancer therapeutic strategies, these data further promote PLAC1 as a promising anticancer drug target.

## INTRODUCTION

Placental trophoblasts and cancer cells exhibit shared characteristics. Both cell types are motile, migratory, and exhibit immune tolerance mechanisms to evade as well as modify the host’s immune response [1]. Proteins typically associated with human embryonic or fetal development are reactivated in cancer cells [2, 3], and may confer tumor cells with invasive potential.

One placental protein that is highly expressed in a wide range of human tumors is placenta enriched 1 (PLAC1) [4]. PLAC1 expression is limited to differentiated cells of the syncytiotrophoblast, with no substantial presence in other tissues [4, 5]. With malignant transformation, PLAC1 is frequently activated and highly expressed in various tumor types, especially in breast cancer [6–9] and prostate cancer [10]. Inhibition of PLAC1 in cancer cells leads to cell cycle arrest via a reduction in AKT phosphorylation and cyclin D levels [11]. The expression of PLAC1 in cancer cells also induces cellular and humoral immune responses via activation of cytotoxic T cells and antibody responses to antigen-presenting cells that carry PLAC1-derived peptides, which leads to the elimination of PLAC1-positive cancer cells [11].

Previously, we reported that PLAC1 is linked to the PI3K/AKT pathway and is critical for motility, migration, and invasion of breast cancer cells [4]. However, to date, the role of PLAC1 in this tumor-promoting pathway is not clearly understood. Since activation of the PI3K/AKT pathway usually results from receptor tyrosine kinase (RTK) activity, we hypothesized that an RTK is involved in PLAC1-mediated activation of the PI3K/AKT pathway. Various reports indicate association of PLAC1 with the fibroblast growth factor receptor (FGFR) family. FGFRs are prominent RTKs that regulate placental trophoblast development [12] and are altered in cancer [13]. The FGFR family consists of four high-affinity FGFRs that are alternatively spliced to generate seven tissue-specific isoforms that bind to the spectrum of 18 mammalian fibroblast growth factors (FGFs) [14]. Aberrant activation of FGFR signaling, stemming from *FGFR* gene mutations and overexpression of FGFRs or their ligands, has been observed in a variety of human tumors [15]. During placental development, several growth factor–mediated signaling pathways regulate proliferation, invasion, and migration of trophoblasts [16]. Signaling by FGFs has diverse cellular consequences that include proliferation, growth arrest, differentiation, and apoptosis [17]. Several FGFs, including FGF4 and FGF7, activate the PI3K/AKT pathway [18, 19]. FGF7, an FGFR2-specific ligand involved in trophoblast proliferation and differentiation, was shown to co-localize with PLAC1 in the placental syncytiotrophoblast [20] and to regulate PLAC1 expression [5, 16]. Based on these observations, it was hypothesized that a placental PLAC1-FGF7 axis regulated trophoblast development via paracrine mechanisms [21, 22]. However, the molecular function of the PLAC1-FGF7 axis in placental development and cancer remains unknown.

This study investigated and characterized the link between PLAC1 and the FGF7/FGFR2IIIb signaling axis, and evaluated the potential role of PLAC1 in tumor cells. Specifically, we characterized the extracellular localization of PLAC1 and its interaction with the FGF7/FGFRIIIb signaling axis using high-resolution microscopy and biochemical binding assays. We evaluated the role of PLAC1 in tumor cells using PLAC1 knockdown and cell signaling assays.

## RESULTS

### PLAC1 is co-expressed with FGF7 and FGFR2 in placenta and human cancer cells and is localized in the ECM

**Figure 1:**
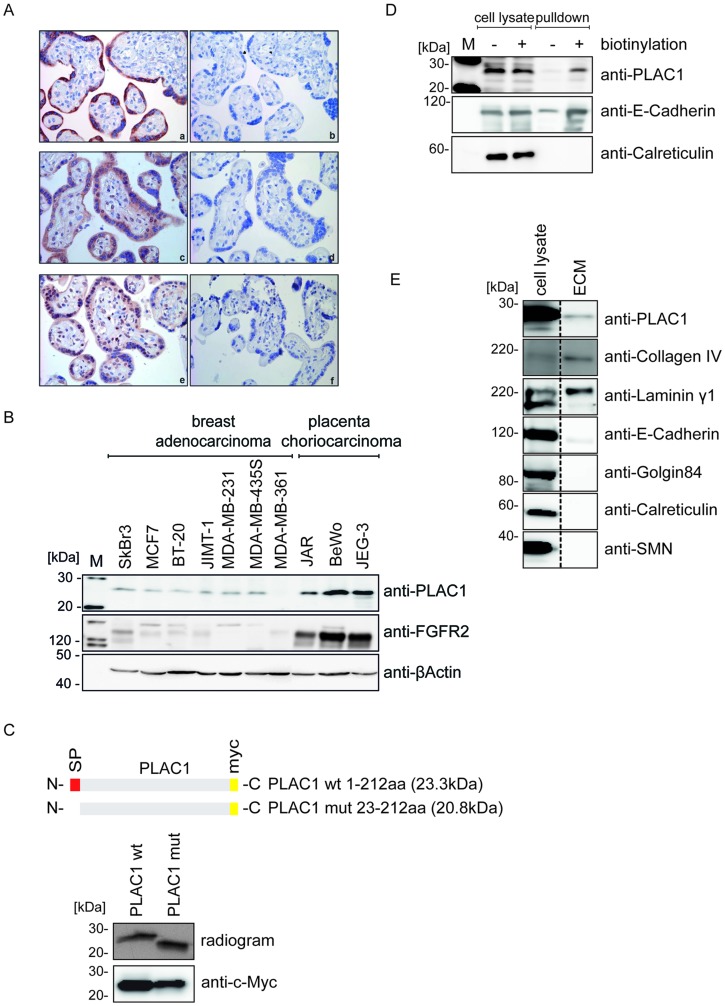
PLAC1 is co-expressed with FGF7 and FGFR2 in placenta and human cancer cells and is released into the ECM. (**A**) Immunohistochemical staining of sections from placental syncytiotrophoblast tissue with anti-PLAC1 (a), anti-FGFR2 (c), and anti-FGF7 (e) antibodies; negative control tissue sections were not treated with the primary antibody (b, d, f; magnification: 40×). (**B**) Protein expression of PLAC1 and FGFR2 was analyzed by Western blotting in breast adenocarcinoma and placental choriocarcinoma cell lines. (**C**) Wild-type (wt) and mutated (mut) PLAC1 constructs were analyzed by sodium dodecyl sulfate–polyacrylamide gel electrophoresis (SDS-PAGE) and autoradiography after coupled *in vitro* transcription and translation (upper panel) or by Western blotting of transfected HEK293T cell lysates (lower panel). (**D**) NeutrAvidin pulldown assays of biotinylated and non-biotinylated BeWo cell surface proteins. Pulldown samples and crude cell lysate were subjected to Western Blot analysis. (**E**) Isolated ECM fractions from BeWo and crude cell lysates were analyzed by Western blotting using antibodies against ECM proteins.

First, we studied the expression of PLAC1, FGFR2, and FGF7. Immunohistochemical staining of placental tissue sections showed strong expression of PLAC1, FGFR2, and FGF7 in the syncytiotrophoblast, confirming previous reports [20] of co-expression of all three proteins within the same cellular structures (Figure 1A). We then screened human cancer cell lines for PLAC1 and FGFR2 expression by Western Blot analysis. Placental choriocarcinoma cell lines with high expression of PLAC1 also showed high levels of FGFR2, whereas the tested breast carcinoma cell lines had low or barely detectable levels of both proteins (Figure 1B; the expression of FGFR2 in T-47D cells is shown in Supplementary Figure 1). To study the subcellular localization of PLAC1, we performed a series of experiments. Sequence analysis predicted an N-terminal signal peptide, implying that PLAC1 may be a secreted protein. We assessed this hypothesis by *in vitro–* and *in vivo–*coupled translation of wild-type PLAC1 and a PLAC1 variant deleted for this predicted signal peptide. Unlike *in vivo* transfection where proteins undergo normal cellular processing, which includes post-translational modifications, *in vitro–*translated proteins are not further processed after translation and reflect the original size of the translated protein. Comparison of the molecular weights of the translated proteins indicated the existence of a cleavable signal peptide (Figure 1C). To further verify the secretion of PLAC1, we isolated the cell surface protein fraction by biotin-labeling of intact BeWo cells followed by NeutrAvidin pulldown and analyzed by Western blotting with a monoclonal anti-PLAC1 antibody. A strong signal was detected in the biotinylated fraction, indicating that PLAC1 is a cell surface protein (Figure 1D). Next, we isolated the extracellular matrix (ECM) by hypotonic lysis of BeWo cells. We verified purity of the ECM preparation by testing for removal of indicator molecules for other cellular compartments. Analysis of ECM samples by Western blotting with a monoclonal anti-PLAC1 antibody revealed the presence of the PLAC1 protein together with collagen IV and laminin γ1 in the ECM (Figure 1E). In summary, our data confirmed co-expression of PLAC1 and FGFR2 in both the placental syncytiotrophoblast and in human cancer cell lines, and demonstrated that PLAC1 is a secreted protein, which accumulates in the ECM.

### PLAC1 forms a trimeric complex with FGF7 and FGFR2IIIb *in vivo*

**Figure 2:**
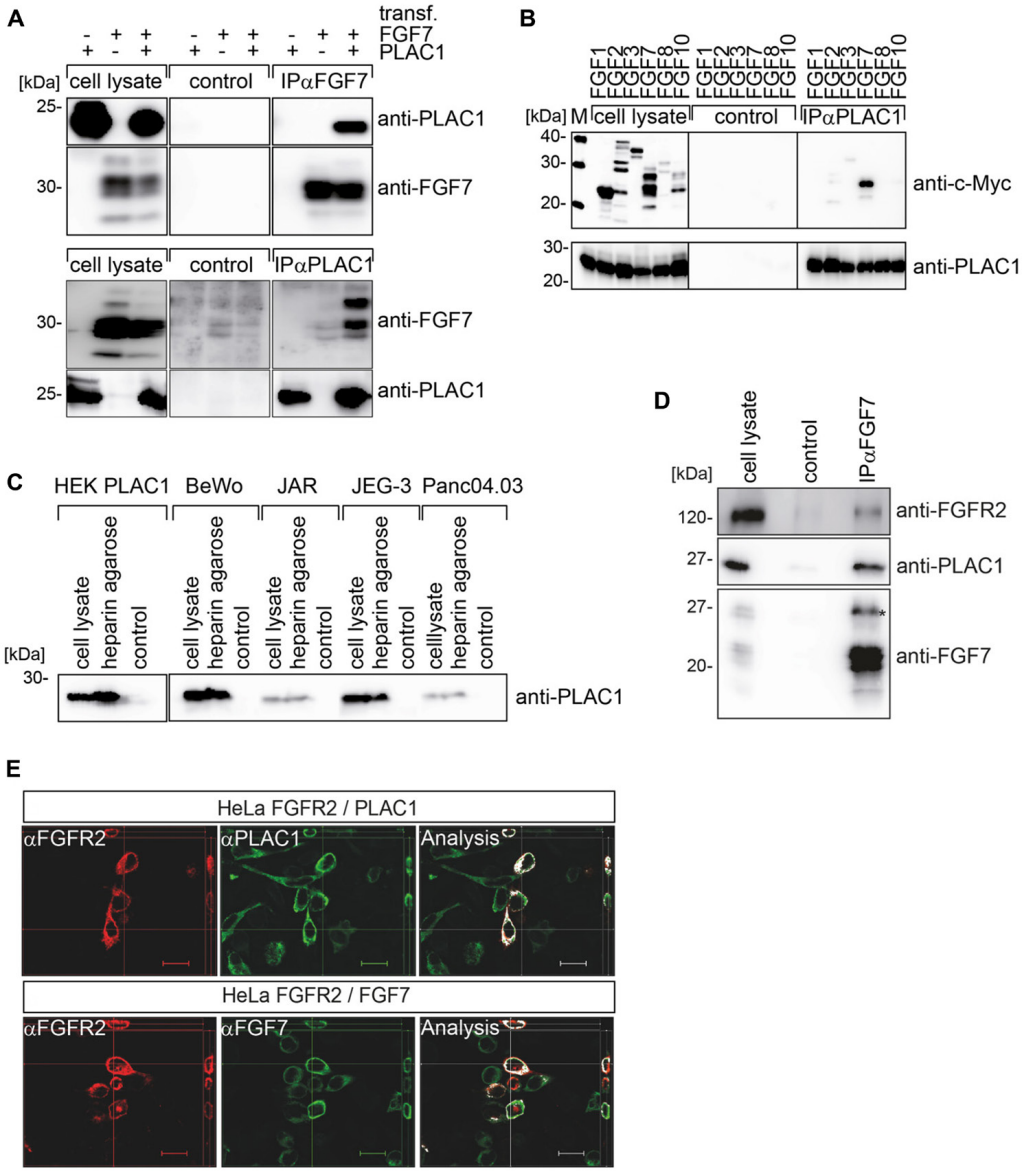
PLAC1 forms a trimeric complex with FGF7 and FGFR2IIIb *in vivo*. (**A**) Results from co-immunoprecipitation assays of FGF7 or PLAC1 from transiently transfected HEK293T cells are shown. FGF7 or PLAC1 was immunoprecipitated from cell extracts with FGF7- (upper panel) or PLAC1-specific antibodies (lower panel), with corresponding IgG antibodies as controls. Immunoprecipitates and crude cell lysates were resolved by SDS-PAGE and analyzed by Western blotting with anti-FGF7 and anti-PLAC1 antibodies. (**B**) Results from co-immunoprecipitation assays of HEK293T cells co-transfected with PLAC1 and six myc-tagged members of the FGF-protein family are shown. Mouse serum was used as control for nonspecific binding. Immunoprecipitates and crude cell lysates were analyzed by Western blotting: FGF (upper panel) and PLAC1 (lower panel). (**C**) Results from heparin pulldown assays of PLAC1 in extracts of transfected HEK293T cells and in cells that endogenously express PLAC1 are shown. The binding of PLAC1 to heparin was assessed by Western blotting using an anti-PLAC1 antibody. (**D**) The FGF7, PLAC1, and a rabbit-IgG-FGFR2IIIb (D1-D3) fusion proteins were co-expressed in HEK293T cells. FGF7 was immune-precipitated from cell extracts with the respective antibodies (lower panel); immobilized IgG served as a control. Immuno-precipitates were resolved by SDS-PAGE and analyzed by Western blotting with antibodies against FGF7, PLAC1, and FGFR2IIIb; the red asterisk represents glycosylated FGF7 isoforms. (**E**) Co-transfected HeLa cells with FGFR2IIIb and PLAC1 (upper panel) or FGFR2IIIb and FGF7 (lower panel) were stained with the corresponding antibodies and analyzed by z-stack-immunofluorescence microscopy. Co-localization analysis was performed using the co-localization plugin of ImageJ. Scale bar = 20 μm.

To directly assess whether PLAC1 and FGF7 interact, co-immunoprecipitation assays were performed. Lysates of PLAC1 and/or FGF7-transfected HEK293T cells were used for immunoprecipitations with anti-FGF7 as well as with anti-PLAC1 antibodies, and captured proteins were visualized through Western Blot by the corresponding antibodies. These assays clearly demonstrated an interaction between PLAC1 and FGF7 (Figure 2A). Due to the highly homologous nature of FGFs, we assessed whether PLAC1 also interacts with other members of the FGF family. We tagged six different FGFs at the C-terminus with a myc-tag and co-transfected each one with PLAC1 into HEK293T cells. Co-immunoprecipitation with an anti-myc antibody was performed and PLAC1 co-immunoprecipitated complexes were analyzed by Western blotting. We observed that only FGF7 was bound to PLAC1, indicating a highly specific interaction (Figure 2B).

Binding and activation of FGFRs is dependent on heparan sulfate glycosaminoglycans (HSGAGs) in the ECM [5]. Due to the localization of PLAC1 in the ECM and its interaction with FGF7, we hypothesized the putative binding of PLAC1 to cell-surface/ECM HSGAGs. Extracts of HEK293T cells transfected with PLAC1 were used in heparin pulldown assays with heparin cross-linked to agarose and mock agarose as negative control. We found that PLAC1 was precipitated by heparin-agarose, but not by the mock-agarose (Figure 2C). To confirm the direct interaction between PLAC1 and HSGAGs in cells that endogenously expressed PLAC1, cell extracts of choriocarcinoma cell lines BeWo, JEG-3, and JAR, and the pancreatic cancer cell line Panc 04.03, were used in heparin pulldown assays. We observed that endogenous PLAC1 was also precipitated by heparin-agarose, but not by mock-agarose (Figure 2C). In addition, PLAC1 also exhibited a strong affinity to heparin and heparan sulfate (HS; data not shown).

Next we addressed the nature of the interaction of PLAC1 with the FGFR2IIIb/FGF7 receptor-ligand complex. We transfected HEK293T cells with FGF7, PLAC1, and a soluble FGFR2IIIb-IgG fusion construct containing the extracellular D1-D3 region of FGFR2IIIb (aa 22-358), and performed FGF7-specific immunoprecipitation assays from cell extracts. Western blotting clearly showed that PLAC1 and the FGFR2IIIb-fusion protein co-precipitated with FGF7 (Figure 2D). Non-specific binding to the IgG control antibody was not observed. To verify the presence of a physiologically expressed trimeric complex, the co-localization of PLAC1 and FGF7, and FGFR2IIIb and FGF7 at the plasma membrane, was confirmed by immunofluorescence microscopy (Figure 2E). HeLa cells were co-transfected with FGFR2IIIb and PLAC1 (Figure 2E, upper panel) or FGFR2IIIb and FGF7 (Figure 2B, lower panel). Merged microscopic images indicated co-localization of PLAC1 and FGFR2IIIb, as well as FGF7 and FGFR2IIIb at the plasma membrane of double-positive HeLa cells. These data therefore provide evidence of a physiologically expressed PLAC1-FGFR2IIIb-FGF7 trimeric complex, which suggest that PLAC1 may be a co-factor of the FGF7/FGFR2 signaling pathway.

### PLAC1 activates AKT phosphorylation in cancer cell lines via FGFR2IIIb signaling and mediates proliferation

**Figure 3:**
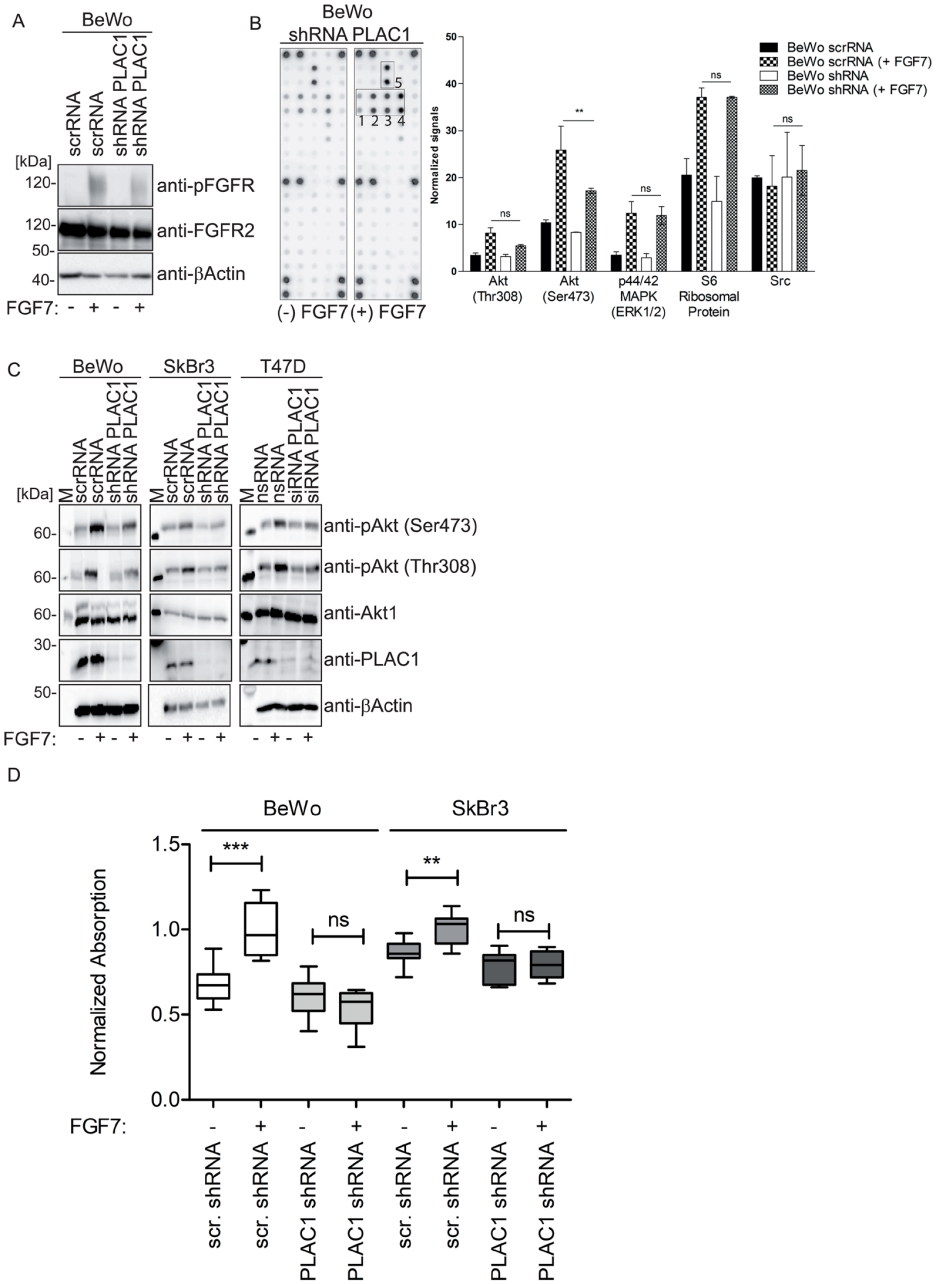
PLAC1 activates AKT phosphorylation in breast cancer and placental cells via FGFR2IIIbR signaling and mediates proliferation. (**A**) The phosphorylation of FGFR2 was analyzed in PLAC1 shRNA or scrambled shRNA-transduced BeWo cells treated with/without FGF7 (200 ng/ml) by Western blotting with anti-FGFR2 and anti-phoshpo-FGFR antibodies. (**B**) Cell extracts of FGF7-stimulated PLAC1-knockdown BeWo cells were evaluated using the PathScan^®^ RTK Signaling Antibody Array Kit to detect downstream targets of PLAC1/FGF7 signaling. Spot intensities were quantified using an array analysis software. Results from densitometry analysis of phosphorylated proteins in FGF7-treated PLAC1-shRNA–transduced BeWo cells and FGF7-treated scrambled shRNA-transduced BeWo cells are shown (data presented as mean ± standard error of the mean; ^**^ *P* ≤ 0.01, *N* = 3). The strongest phosphorylated receptor tyrosine kinases are highlighted in the boxes numbered 1 to 5 (1: spot #29 - Akt Ser473; 2: spot #30 - Akt Thr308; 3: spot #31- p44/42 MAPK[ERK1/2]; 4: spot #32 - S6 ribosomal protein; 5: spot #36 – Src). (**C**) AKT phosphorylation in cell extracts of FGF7-stimulated PLAC1-knockdown BeWo, SkBr3, and T47D cells was analyzed by SDS-PAGE and Western blotting with anti-AKT and anti-phospho-AKT antibodies. (**D**) The proliferation of BeWo and SkBr3 cells or PLAC1 shRNA or scrambled (scr) RNA-transduced cells was measured using XTT after FGF7 stimulation (200 ng/ml) and normalized to the control group (^**^ *P* ≤ 0.01, ^***^ *P* ≤ 0.001, whiskers represent 5-95 percentile).

To assess whether PLAC1 is a co-factor of the FGF7-FGFR2 signaling pathway, we analyzed PLAC1-dependent activation of FGFR2. Knockdown assays of *PLAC1* gene expression in BeWo cells were performed by lentiviral transduction using a short hairpin RNA (shRNA) against PLAC1 or a scrambled shRNA with or without subsequent FGF7 treatment, and the phosphorylation status of FGFR2 was analyzed. We verified the efficiency of PLAC1 knockdown in shRNA-transduced BeWo cell lysates by Western blotting using β-actin as a control (Supplementary Figure 2). Western Blot analysis of cell lysates revealed that FGFR2 phosphorylation was markedly reduced after PLAC1 knockdown in cells stimulated with FGF7 (Figure 3A); FGFR2 phosphorylation was not observed in non-stimulated cells (Figure 3A). A PathScan^®^ RTK Signaling Antibody Array was used (Figure 3B) to detect intracellular signaling networks mediated by PLAC1 in FGF7-stimulated PLAC1-knockdown BeWo cell extracts. Phosphorylation of AKT at Ser473, mitogen-activated protein kinase (MAPK), S6, and Src was observed; however, only the phosphorylation of AKT at Ser473 was significantly reduced after PLAC1 knockdown (Figure 3B).

Further analysis of FGF7-stimulated PLAC1-knockdown BeWo cells showed that FGF7-mediated phosphorylation of AKT at Ser473 and Thr308 was markedly reduced in the absence of PLAC1 compared with control cells (Figure 3C). Additionally, similar effects of FGF7 stimulation on AKT phosphorylation following PLAC1 knockdown in breast cancer cell lines SkBr3 (Figure 3C) and T47D (Figure 3C) were shown. The substantial decrease in AKT phosphorylation after PLAC1 knockdown suggested that PLAC1 is involved in FGF7-induced AKT phosphorylation via the FGFR2IIIb receptor.

Because AKT activation leads to increased cell proliferation [23, 24], we evaluated whether PLAC1, as a co-factor of the FGF7-FGFR2 signaling pathway, also mediated cancer cell proliferation. We compared proliferation before and after PLAC1 shRNA transduction in FGF7-stimulated BeWo (Figure 3D, left panel) and SkBr3 cells (Figure 3D, right panel) to demonstrate PLAC1 dependent proliferation.

Overall, these results show that cellular proliferation is dependent on the presence of both PLAC1 and FGF7, which formed a complex with FGFR2IIIb and induce cell proliferation by activation of AKT.

## DISCUSSION

**Figure 4:**
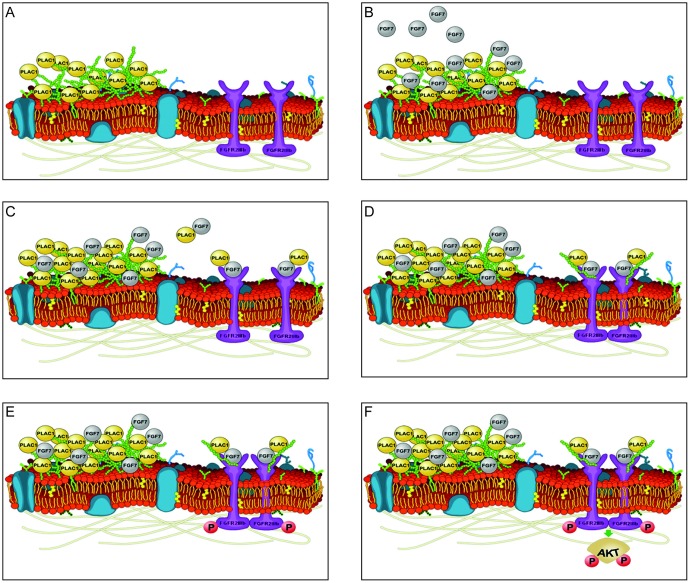
Model of PLAC1-mediated FGF signaling. (**A**) Secreted PLAC1 forms stable multimers at the cell surface that interact with components of the ECM (e. g. heparin). (**B**) Paracrine-secreted FGF7 binds to the ECM due to its strong affinity for glycosaminoglycans (GAGs) and PLAC1. (**C**) Interacting PLAC1-FGF7 molecules are released from the ECM and bind to FGFR2IIIb. (**D**) Binding of the PLAC1-FGF7 complex together with the GAG moiety promotes dimerization of FGF2Rs. (**E**) Dimerization of the FGFR2IIIb-PLAC1-FGF7 complex in turn activates the intracellular tyrosine kinase of FGFR2IIIb via trans-phosphorylation. (**F**) The active kinase domain mediates signal transduction through direct phosphorylation of adaptor proteins and by phosphorylation of sites on the receptor that act as docking sites for other adaptor proteins [17, 25, 26]. Activated adapter proteins promote recruitment and activation of AKT, which mediates cell survival, proliferation, and migration by binding to and regulating several downstream targets [27, 28]. Images of cell membranes were adapted from Wikimedia Commons (permission to use the image for any purpose was granted by the image creator, Mariana Ruiz prior to the inclusion of the image in the manuscript).

This study provides new insights into the role of PLAC1 in tumor cells and in placental syncytiotrophoblasts. We demonstrate firsthand the potential role of PLAC1 in the tumorigenesis of breast cancer cells and choriocarcinoma cells via formation of a trimeric complex between PLAC1, FGF7, and FGFR2IIIb, which binds to HSGAGs in the ECM, and leads to cell proliferation via the activation of AKT. These findings establish a new model of FGF signaling in tumor cells (Figure 4). In normal placental development, signaling via the PLAC1-FGF7 axis likely mediates the proliferation and migration of syncytiotrophoblasts.

We demonstrated the release of PLAC1 into the ECM and its association with HS and heparin. Both HS and heparin are highly similar in structure and typically bind to FGFs and FGFRs, facilitating the formation of a ternary complex consisting of two heparin moieties, two FGF molecules, and two FGFRs [29, 30]. Within this complex, heparin promotes dimerization of the ligand-receptor complex, whereby FGFs mediate cellular effects by activating the intracellular receptor tyrosine kinase through trans-phosphorylation [15, 17, 25]. Another role of HS binding might be the creation of a local growth factor reservoir, which allows for tight spatial regulation of FGF signaling that is generally restricted to cells in contact with the ECM [17]. Thus, the binding of PLAC1 to heparin in the ECM may create a localized PLAC1 reservoir that surrounds the cell and allows for optimal positioning of PLAC1 within the ligand-receptor complex.

In placental choriocarcinoma and breast cancer cells, we demonstrated that among members of the FGF family, PLAC1 exerts its function exclusively through FGF7, as we did not observe PLAC1 binding to other FGFs. As such, FGF7, also known as keratinocyte growth factor, is produced by cells of mesenchymal origin and acts only through the FGFR2IIIb isoform that is primarily expressed by epithelial cells [31]. Notably, FGF7 plays an important role in regulating the growth of the mammary epithelium [32] and also contributes to mammary tumorigenesis, a cancer type in which PLAC1 is found to be frequently expressed [33]. Indeed, constitutive overexpression of FGF7 in the mammary epithelium of transgenic mice induces mammary hyperplasia and adenocarcinoma [34, 35].

Key intracellular pathways associated with FGF signaling are the RAS-MAPK and PI3K/AKT pathways. Activation of the RAS-MAPK and PI3K/AKT pathways lead to different cellular responses depending on cell type or differentiation stage [15]. We found three intracellular targets with FGF7 stimulation of BeWo cells: S6, MAPK, and AKT. PLAC1 knockdown resulted in a significant reduction of FGF7-induced phosphorylation of AKT, but not S6 or MAPK, which suggests that PLAC1 contributes to FGF7-mediated activation of the PI3K/AKT pathway, but not the RAS-MAPK pathway.

AKT mediates proliferation by regulating cell-cycle progression [36]. In breast cancer, it was shown that cyclin D1 expression, the major regulator of the G1-S progression of the cell cycle, was controlled by PIK-3/AKT signaling [37, 38]. We have previously shown that cyclin D1 expression and AKT phosphorylation are markedly reduced in breast cancer cell lines after PLAC1 knockdown [4]. Here we extended this function of PLAC1 to placental cells, suggesting the reactivation of a placental pathway in breast cancer.

Findings from our study position PLAC1 in the PI3K/AKT pathway, which is highly relevant for therapeutic intervention [39]. Although mutations in the *AKT* gene are not widely reported, amplification, overexpression, and activation of AKT frequently occurs in a number of cancers [40]. Studies have demonstrated that phosphorylated AKT is associated with ErbB2 overexpression and poor disease-free survival in breast cancer [41, 42]. Several inhibitors of the PI3K/AKT/mTOR pathway are being evaluated in preclinical studies [43, 44]. Of note, we demonstrated that PLAC1 knockdown effectively counteracted FGF7-induced AKT phosphorylation and cell proliferation. Thus, inhibition of PLAC1 may represent an attractive therapeutic intervention. Because PLAC1 knockdown directly targets FGFR2 phosphorylation, which lies upstream of the AKT pathway, inhibition of PLAC1 may effectively counteract FGF7-induced aberrant AKT activation. The effect of PLAC1 knockdown on constitutively active mutant AKT expressed in tumor cells warrants investigation.

In addition to the role of PLAC1 in FGF7-induced AKT activation, there is also evidence to suggest that PLAC1 regulates inflammatory responses and immune tolerance [45]. In EO771 mammary carcinoma cells, knockdown of PLAC1 resulted in the reduced expression of several inflammatory and immune factors, including Cxcl1, Ccl5, Ly6a/Sca-1, Ly6c, and leukemia inhibitory factor [45]. In mice engrafted with wild-type EO771 cells, treatment with a Cxcr2 antagonist inhibited tumor growth, reduced myeloid suppressor and regulatory T cells with concomitant increases in macrophages, dendritic cells, and natural killer cells, and reduced tumor infiltration of CD8+ T cells. Thus, PLAC1 appears to regulate immune tolerance via the chemokine axis [45].

In conclusion, this study demonstrates the contribution of PLAC1 to cell proliferation in placental and tumor tissues. Our results provide the rationale for the clinical development of therapies designed to inhibit PLAC1 function. The role of PLAC1 and its cancer-selective, cell-surface expression make it an attractive candidate for antibody-mediated therapeutic strategies.

## MATERIALS AND METHODS

### Materials

All cell culture media, fetal calf serum (FCS), and supplements were obtained from Invitrogen. Cell culture dishes were purchased from Greiner Bio-One (Kremsmünster, Austria). Purified human FGF7 was provided by Ganymed Pharmaceuticals AG (Mainz, Germany). Unless otherwise mentioned, all other reagents were obtained from Sigma-Aldrich (Munich, Germany).

### Primary antibodies

PLAC1 antibodies were provided by Ganymed Pharmaceuticals AG. FGF7 antibodies (AF-251-NA and K-4760) were purchased from R&D Systems (Minneapolis, MN, USA) and Sigma Aldrich. Mouse anti-HA.11 (raw ascites fluid) was purchased from Covance (Princeton, NJ, USA), and rabbit anti-c-Myc (A-14) and rabbit anti-FGFR2 (sc-122) were purchased from Santa Cruz Biotechnology (Heidelberg, Germany). Mouse anti-phospho-FGF receptor (Tyr653/654) (55H2;#3476), mouse anti-Calreticulin (FMC75), and rabbit anti-Collagen IV (ab52235) were purchased from Abcam (Cambridge, UK), anti β-Actin (A1978) was purchased from Sigma-Aldrich, mouse anti-Golgin-84 (611383) and mouse anti E-Cadherin (610182) were purchased from BD Biosciences (Franklin Lakes, NJ, USA), mouse anti-Laminin-gamma1 (MAB2139) from R&D Systems, mouse anti-Lamin B (61047C) was purchased from Progen (Heidelberg, Germany), and mouse anti-Akt1 (2H10; #2967), rabbit anti Phospho-Akt (Ser473) (#9271), and rabbit anti-Phospho-Akt (Thr308) (244F9; #4056) were purchased from Cell Signaling Technology (Danvers, MA, USA). The SMN antibody was kindly provided by Matthias Grimmler [46].

### Cloning of cDNA constructs

Plasmids encoding full-length cDNAs corresponding to the open reading frames of human PLAC1, human FGFR2IIIb, as well as human FGF-1, 2, 3, 7, 8, and 10 were purchased as codon-optimized constructs from Geneart (Regensburg, Germany). The full-length open reading frames were subcloned into pGEX6P-1 (GE Healthcare, Freiburg, Germany), pHA (a N-terminal HA-tag containing derivate of pCDNA3.1; Invitrogen, Carlsbad, CA, USA), and pCDNA3.1 (Invitrogen), respectively. Truncations of human PLAC1 as well as FGFR2IIIb fusion constructs were generated by polymerase chain reaction and subcloned into pCR3.1 (Invitrogen) or rather into pFUSE-rabbit IgG (IL2ss) (a derivate of pCDNA3.1 containing a IL2 secretion signal and the heavy chain of rabbit IgG1, Invitrogen).

### Cloning of shRNA construct

The shRNA constructs for the knockdown of human PLAC1 (NCBI Reference Sequence: NM_021796.3) were designed using the “BLOCK-iT™ RNAi Designer” online tool (Life Technologies, Darmstadt, Germany) [47]. Two different best ranked target sequences for shRNA (#59: 5′-ggttcaggacaaagtccaatg-3′; #60: 5′-GCTACGAGGTGTTCAGCTTGT-3′) were chosen and oligonucleotides were designed by following the instructions on the website. Forward and reverse oligonucleotides were annealed to generate double-stranded linkers with overhangs compatible with the commercially available precut BLOCK-iT™ entry vector (pENTR™/U6) (Life Technologies) and ligated into this vector. pENTR5′-CMV was cut using HindIII and BamHI restriction enzymes to excise the CMV promoter. The vector was blunted and re-circularized to generate an empty pENTR5′. To generate a green fluorescent protein (GFP)-tagged lentiviral gateway™ destination vector, the blasticidin resistance gene was removed from pLenti6.4-R4R2-V5-DEST and replaced by enhanced GFP (pLenti6.4-GFP-DEST). Empty pENTR5′, shRNA containing pENTR™/U6, and pLenti6.4-GFP-DEST were recombined using the LR-Clonase^®^ II Plus enzyme kit following the manufacturer’s instructions (Life Technologies) to get lentiviral shRNA vectors.

### Tissues and cell lines

Analysis of protein expression in human tissue and cell lines was performed with permission from, and according to the rules of, the state government of Rheinland-Palatinate. Tissues were obtained as human surplus materials during routine diagnostic or therapeutic procedures and were stored at –80°C until the time of use. All cell lines were obtained from American Type Culture Collection (Manassas, VA, USA), cultured in appropriate media at 37°C and either 7.5% CO_2_ (HEK293T cells) or 5% CO_2_ (all other cell types), and were passaged for less than 6 months after receipt.

### Transient cDNA transfection of cell lines

For transfection experiments, HEK293T were seeded on 10-cm dishes in antibiotic-free media and cultivated for 24 hours. cDNA constructs (24 μg) were supplemented with 80 μl Polyethylenimine (pH 7.0, 1 mg/ml) in serum-free media and incubated for 30 minutes. Subsequently, the mixture of cDNA/PEI was added drop-wise to the plates and the cells were incubated for 48 hours. The medium was changed 8 hours post-transfection. BeWo and HELA cells were transfected using Lipofectamin LTX (Invitrogen) according to manufacturer’s instructions.

### Transient RNA interference

The cellular levels of PLAC1 in SkBr3 and T47D were reduced by transfection of a mixture of two double-stranded 21-nucleotide–long siRNAs (sequences: 5′-CUCCAUGAGAGUAGCCAGCAA-3′ and 5′- CCGGUUCAGGACAAAGTCCAA-3′, Dharmacon). For *PLAC1* gene-silencing studies, cells were transfected with 10 nM siRNA duplex using HiPerFect transfection reagent (Qiagen, Hilden, Germany) according to the manufacturer’s instructions. Western blotting of cell extracts obtained 48 hours after transfection using PLAC1-specific antibodies was used to confirm PLAC1 gene silencing.

### Lentiviral transduction and stable knockdown

Lentiviral supernatants were derived from HEK293T cells at 7 × 10^4^ cells/cm^2^ in 0.175 ml medium per cm^2^. The next day, cells were transfected with TransIT^®^ LT1 (Mirus Bio LLC; Madison, WI, USA) using 0.63 μl of reagent per cm^2^. The DNA mixtures used were composed of 84 ng/cm^2^ lentiviral vectors, 96 ng/cm^2^ GAG-POL coding pCMVdeltaR8.91, and 32.8 ng/cm^2^ VSV-G coding pMD2G. Twenty-four hours after transfection, the culture medium was renewed and the lentiviral supernatant was harvested at 48 hours and 72 hours after transfection. For transduction, lentiviral supernatants were loaded three repeated times onto Retronectin^®^-coated, non-tissue culture-treated 24-well-plates according to the manufacturer’s instructions (Takara Bio Inc. Shiga, Japan). Thereafter, BeWo cells were seeded at a density of 4 × 10^4^ adherent cells per well and incubated for 48 hours with the viral particles. Successful transductions were assessed by flow cytometry.

### SDS-PAGE and Western blotting

Total cell extracts were generated by scraping cells from the cell culture plate after applying a 4× SDS-lysis buffer (250 mM Tris/HCl, 34% [w/v] Glycerin, 8.2% [w/v] SDS, 5% [v/v] β-Mercaptoethanol, pH 6.8). After sonication, the protein concentration in cell extracts was measured by spectrophotometry at OD280. Equal amounts of protein were loaded onto SDS-PAGE gels and subsequently transferred onto nitrocellulose membranes (0.1 μm, GE Healthcare). Immunostaining was performed with primary antibodies followed by secondary horseradish-peroxidase conjugated rabbit anti-mouse, goat anti-rabbit, rabbit anti-goat secondary antibodies (Pierce Biotechnology, Rockford, IL, USA). Non-specific binding to the membranes was blocked with 5% (w/v) skim milk powder solutions in PBS-T (PBS: 140 mM NaCl, 2.7 mM KCl, 10 mM Na_2_HPO_4_, 1.8 mM KH_2_PO_4_, pH 7.4; 0.05% [w/v] Tween 20). For detection of phosphorylated proteins in signaling analyses, non-specific binding to nitrocellulose membranes was blocked with 5% (w/v) BSA in TBS-T (50 mM Tris, 150 mM NaCl, 0.05% [w/v] Tween 20, pH 7.4). Protein detection by chemiluminescence was performed using the Lumi-Light Western Blotting substrate (Roche, Basel, Switzerland), Dura or Femto reagent (Pierce), and the ImageQuant LAS 4000 detection system (GE Healthcare). Quantification of signal intensity was performed using ImageQuant TL software (GE Healthcare).

### Coupled *in vitro* transcription and translation

To analyze the subcellular localization of PLAC1, an *in silico-*predicted signal peptide, with a cutting site between amino acid (aa) 22 to 23, was designed to produce a truncated form of PLAC1 *in vivo*. *In vitro–*coupled transcription/translation of proteins was performed using the TNT Quick Coupled Transcription/Translation Reticulocyte Lysate System according to the manufacturer´s protocol (Promega, Madison, WI, USA), using radioactive labeled L-[35S] methionine (Hartmann Analytic GmbH, Braunschweig, Germany). Reactions were incubated for 90 minutes at 30°C. Equal amounts of sample were then treated with 4× SDS-lysis buffer and analyzed by SDS-PAGE and autoradiography.

### Isolation of cell surface proteins

Cell surface proteins were isolated by biotin-labeling of intact cells, followed by NeutrAvidin pulldown, and were analyzed by Western blotting using a monoclonal anti-PLAC1 antibody. The Cell Surface Protein Isolation Kit (Pierce Biotechnology) was used according to the manufacturer’s protocol to identify proteins of the cell surface by biotin-labeling and affinity selection. After biotinylation, crude cell lysates were harvested and centrifuged at 20,000 g for 20 minutes at 4°C. Input was taken from supernatant fractions (ie, crude cell lysates) after centrifugation. Clarified supernatants were pre-depleted with a NHS-Sepharose matrix (GE Healthcare), covalently coated with BSA for 1 hour at 4°C. Biotinylated cell surface proteins were isolated by incubation with NeutrAvidin Agarose for 2 hours at 4°C. The eluted protein and flow-through fractions were collected. Various antibodies were used to control for proper removal of cellular compartments (E-cadherin, Golgin84, Calreticulin, Survival of Motor Neurons) and the enrichment of the ECM (collagen IV and laminin γ1).

### ECM isolation

BeWo cells were seeded at a density of 5 × 10^6^ cells per 10-cm dish and cultivated for 48 or 72 hours. Isolation of the ECM fraction was performed as previously described [48]. Briefly, cells were washed twice with Mili-Q water and incubated at 4°C in Milli-Q water containing complete protease inhibitor (Roche). After 2 hours most cells had spontaneously lifted off the dish surface and any remaining adherent cells were removed by washing and incubation with 0.1% deoxycholate for 1 minute under microscopic control. The ECM was then scraped off in 100 μl of 4× SDS-sample buffer.

### Immunohistochemistry analysis

Formalin-fixed and paraffin-embedded human placental tissue sections were de-paraffinized in xylene and rehydrated in descending concentrations of ethanol. The tissue sections were then subjected to antigen retrieval by heating for 10 minutes at 120°C in citrate buffer (pH 6) and allowed to cool down to room temperature. The tissue sections were treated with 0.3% H_2_O_2_ to block endogenous peroxidases and with goat or rabbit serum to block nonspecific antibody binding sites on PLAC1 and FGFR2IIIb or FGF7, respectively. Samples were incubated with primary antibodies against PLAC1 (mouse monoclonal, mu37, Ganymed Pharmaceuticals AG), FGFR2 (rabbit polyclonal, sc-122, Santa Cruz Biotechnology), or FGF7 (polyclonal goat, AF-251-NA, R&D Systems) overnight at 4°C; the sections were washed three times with phosphate-buffered saline (PBS) and then incubated with horseradish peroxidase-conjugated secondary antibodies, goat-anti-mouse, goat-anti-rabbit, or rabbit-anti-goat (Immunologic, bv, Duiven, Netherlands), respectively. For visualization of PLAC1, FGFR2IIIb, and FGF7 localization, the Vector NovaRED peroxidase substrate kit (Vector Laboratories Inc. Peterborough, UK) was used and nuclei were counterstained with hematoxylin/eosin. Tissue sections were dehydrated and mounted (X-TRA-Kitt, Medite, Burgdorf, Germany) prior to microscopic evaluation.

### Immunofluorescence analysis

HeLa cells were seeded onto cover slides. After 24 hours, cells were co-transfected with PLAC1, FGF7, and FGFR2IIIb constructs. After incubation for 48 hours, cells were washed twice in PBS, fixed with 4% paraformaldehyde (in PBS) for 10 minutes at room temperature, and permeabilized with 0.5% triton X-100 (in PBS). After blocking cells with 5% bovine serum albumin (BSA; in PBS), cells were incubated for 1 hour at 37°C with a combination of mouse anti-PLAC1, goat or mouse anti-FGF7, and rabbit anti-FGFR2IIIb antibodies in 0.5% BSA (in PBS) at 37°C. Following staining with combinations of rabbit αGoat Cy3, goat αMouse Cy2, and Goat αRabbit Cy3 (Jackson ImmunoResearch Laboratories, West Grove, PA, USA), cells were evaluated under a Zeiss Axio Imager. M2 microscope with a 40× oil immersion lens. Microscopic images were analyzed using the Image J Co-localization plugin (default settings) [49].

### Co-immunoprecipitations and HSGAG-pulldown

For soluble native protein extracts, cells were carefully removed from cell culture dishes by washing with PBS, and cell pellets were generated by centrifugation (8 minutes, 1200 rpm, 4°C). Pellets were resuspended in lysis buffer (PBS, 0.01% [w/v] Triton X-100, 2 mM EDTA, 2 mM EGTA, 5% [v/v] β-Mercaptoethanol, complete protease inhibitor [Roche], pH 7.5), cells were lysed by sonication, and high-speed centrifugation (21000 g, 30 minutes, 4°C) was used to obtain purified supernatant fractions. For co-immunoprecipitation assays, lysates of PLAC1- or FGF7-transfected HEK293T cells were used in combination with anti-FGF7 and anti-PLAC1 antibodies. Antibodies were coupled to protein A or NHS-sepharose (Sigma-Aldrich, GE Healthcare) according to manufacturer’s instructions. Antibody-coupled beads were equilibrated twice with 0.2 M Na-tetraborate, pH 9.0 and cross-linked with 0.052 g dimethylpimelimidate (DMP) in 10 ml borate-buffered DMP for 30 minutes at room temperature. The cross-linking process was stopped with 50 mM Tris/HCl, 5.2 M NaCl, 0,01% (w/v) Triton X-100, pH 7.0. Coupled antibodies or HSGAG beads were incubated with native protein extracts for 2 hours at 4°C on a rotating mixer. Precipitated protein complexes were washed extensively with PBS pH 8.5, 0.01% Triton-X 100, 2 mM EDTA, and 2 mM EGTA, and eluted by boiling in 4× SDS sample buffer. Immunoprecipitated protein samples were resolved by SDS-PAGE and analyzed by Coomassie staining or Western blotting.

### Signaling analysis: generation of FGF7-stimulated lysates

At 48 hours after seeding BeWo or SkBr3 cells with stable PLAC1 shRNA expression, or at 48 hours after transient PLAC1 knockdown in T47D, cells were incubated with growth medium containing 2% fetal calf serum (FCS) for 4 hours. Cells were then stimulated with 200 ng/ml FGF7 for 30 minutes, washed with PBS, and harvested in the appropriate lysis buffer for the signaling array kit according to manufacturer’s instructions. The lysis buffers were supplemented with complete protease inhibitor, PhosSTOP (Roche), and 1 mM NaVO_4_. For Western blotting analysis, 4× SDS-lysis buffer was added to samples.

### Signaling analysis: array analysis

The PathScan^®^ RTK Signaling Antibody Array Kit (Cell Signaling Technology) was used according to the manufacturer’s instructions. Phosphorylated signaling proteins were detected via chemiluminescence using the Dura reagent (Pierce) and the ImageQuant LAS 4000 detection system (GE Healthcare). Quantification of signal intensity was performed via the Image Quant TL software (GE Healthcare).

### XTT assay

Cell proliferation was assessed using the Cell Proliferation Kit II (XTT, Roche). Stably transduced BeWo and SkBr3 cells were cultured at low (2%) FCS concentrations for 24 hours. Cells were subsequently stimulated with FGF7 (200 ng/ml) for 24 hours. Proliferation was measured using XTT according to the manufacturer´s protocol.

### Statistical analysis

Data analysis was performed using the Graph Pad Prism program (version 5.01, Graph Pad software). Results were reported as the arithmetic mean and standard deviation. The analysis of variance was used to determine differences between group means.

## SUPPLEMENTARY MATERIALS



## Abbreviations

BSA: Bovine serum albumin
CMV: Cytomegalovirus
DMP: Dimethylpimelimidate
DNA: Deoxyribonucleic acid
ECM: Extracellular matrix
EDTA: Ethylenediaminetetraacetic acid
EGTA: Ethylene glycol tetraacetic acid
FCS: Fetal calf serum
FGF: Fibroblast growth factor
FGF7: Fibroblast growth factor 7
FGFR: Fibroblast growth factor receptor
FGFR2: Fibroblast growth factor receptor 2
FGFR2IIIb: Fibroblast growth factor receptor 2 IIIb
GFP: Green fluorescent protein
HSGAG: Heparin sulfate glycosaminoglycan
MAPK: Mitogen-activated protein kinase
NHS: N-hydroxysuccinimide
PBS: Phosphate-buffered saline
PBS-T: Phosphate-buffered saline–Tween 20
PI3K: Phosphoinositide 3-kinase
PLAC1: Placenta enriched 1
scrRNA: Scrambled ribonucleic acid
SDS-PAGE: Sodium dodecyl sulfate polyacrylamide gel electrophoresis
shRNA: Small hairpin ribonucleic acid
siRNA: Small interfering ribonucleic acid
