# Supplementary material for: PLAC1 is essential for FGF7/FGFRIIIb-induced Akt-mediated cancer cell proliferation
Source: Oncotarget. 2020 May 19;11(20):1862–75. doi: 10.18632/oncotarget.27582 (PMC7244013; doi:10.18632/oncotarget.27582)
Supplement: Supplementary file 1 [file oncotarget-11-1862-s001.pdf]

PLAC1 is essential for FGF7/FGFRIIIb-induced Akt-mediated cancer cell proliferation

SUPPLEMENTARY MATERIALS

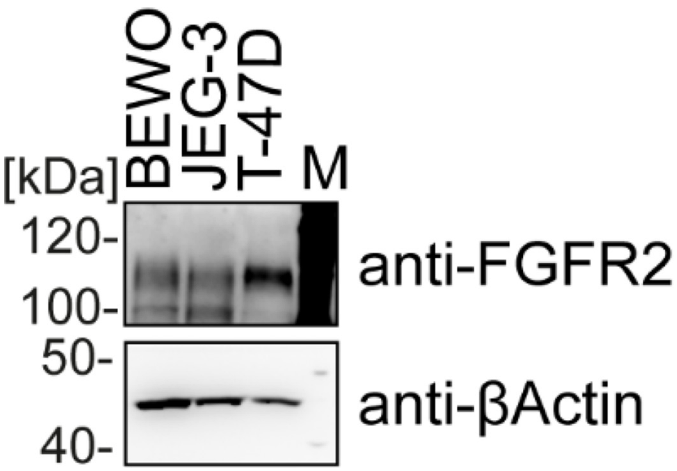

Supplementary Figure 1: Expression of FGFR2 in human cancer cell lines. The expression of FGFR2 protein was analyzed by Western blotting in breast adenocarcinoma and in placental choriocarcinoma BeWo, JEG-3, and T47D cell lines. Abbreviation: FGFR2, fibroblast growth factor receptor 2.

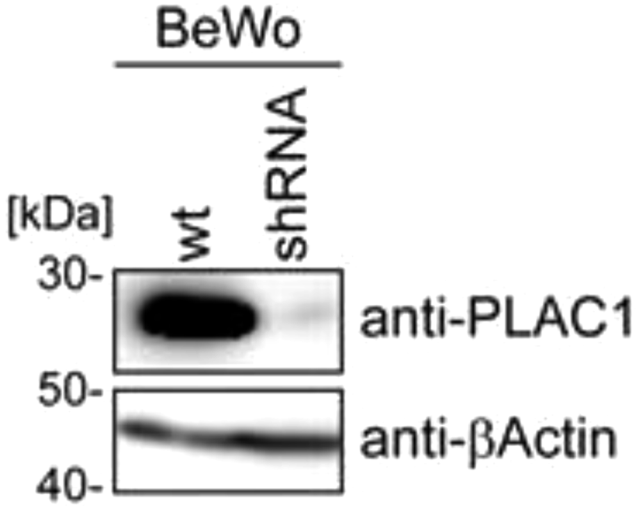

Supplementary Figure 2: Expression of PLAC1 in BeWo cells after shRNA-mediated PLAC1 knockdown. The knockdown of PLAC1 by shRNA by Western blotting was assessed in BeWo cell lysates. Abbreviations: PLAC1, placenta-enriched 1; shRNA, short hairpin RNA; wt, wild-type.
